# Editorial: Regulation of alternative splicing in plant stress responses

**DOI:** 10.3389/fpls.2022.1120961

**Published:** 2023-01-17

**Authors:** Kranthi K. Mandadi, Ezequiel Petrillo, Alexandra S. Dubrovina, Konstantin V. Kiselev

**Affiliations:** ^1^ Department of Plant Pathology and Microbiology, Texas A&M University, College Station, TX, United States; ^2^ Texas A&M AgriLife Research and Extension Center, Texas A&M University System, Weslaco, TX, United States; ^3^ Institute for Advancing Health Through Agriculture, Texas A&M AgriLife, College Station, TX, United States; ^4^ Instituto de Fisiología, Biología Molecular y Neurociencias (IFIBYNE), Consejo Nacional de Investigaciones Científicas y Técnicas (CONICET)-Universidad de Buenos Aires, Buenos Aires, Argentina; ^5^ Federal Scientific Center of the East Asia Terrestrial Biodiversity, Far Eastern Branch of the Russian Academy of Sciences, Vladivostok, Russia

**Keywords:** alternative splicing, post-transcriptional regulation, plant growth and development, abiotic stress, biotic stress

Plants are sessile organisms capable of adaptation to various environmental constraints, such as temperature stress, drought, salinity, and/or pathogen attack. To survive unfavorable conditions, plants actively regulate the expression of stress-responsive genes and transcripts. Alternative splicing is a regulatory process where pre-mRNA is variably spliced, generating several transcripts from a single gene, and expanding genome capacity. In this manner, different mRNA transcripts can lead to the synthesis of several structurally and functionally distinct protein isoforms or products, amplifying the diversity of a plant proteome and reprogramming intracellular regulatory networks using a limited number of genes. Recent genome-wide studies revealed that alternative splicing is highly pervasive in plants, with at least 40-60% of intron-containing genes producing different isoforms. This highlights the importance of alternative splicing in plant performance, adaptation, and survival. The purpose of this Research Topic was to collate the most recent advances in plant alternative splicing research, from identifying alternative splicing events to investigating the functions of specific splicing factors involved. A total of 6 manuscripts, including original research and reviews, were accepted and published.

Liu et al. reviewed and cataloged different stress-responsive alternative splicing events in plants. The authors also provide a summary of the different levels of alternative splicing regulation occurring through splicing factors, epigenetic modifications, shared target binding, and adjustment of splice-variant ratios. Post-translational modifications (PTMs) such as phosphorylation and ubiquitination are critical regulators of alternative splicing. Lan et al. reviewed the role of ubiquitin and ubiquitin-like modification of spliceosome components and how they influence plant development and stress responses. Together, these reviews provide readers with an overview of alternative splicing events and their regulation during plant growth, development, and stress responses.

Advances in next-generation sequencing technologies have greatly enabled studies of plant alternative splicing, particularly in non-model plant species. Ruggiero et al. evaluated global transcriptomic and alternative splicing changes occurring among two contrasting tomato genotypes that were either tolerant or susceptible to water deficit stress, low nitrate stress, or a combination of both. Several genes in the TOR pathway, phytohormone metabolism, transport and signaling, abscisic acid, ethylene, and auxin-related pathways, are differentially spliced under stress conditions. Many affected transcripts encode MYB, basic Helix-Loop-Helix, basic Leucine Zipper, WRKY family transcription factors, Heat Shock Proteins, Auxin Response Factors, and Serine/Arginine-rich (SR)-splicing factors. Further functional characterization of these alternative splicing events could enable the development of superior tomato cultivars tolerant to environmental stresses and limited inputs.

Not only are alternative splicing decisions regulated by stress, but they also impact plant growth and development. Similar to human-splicing-associated disorders, perturbations of normal splicing can result in deleterious and lethal phenotypes in plants. Xu et al. identified one such rice *albino leaf4* mutant (*al4*) that is associated with the activation of an exon-skipping (ES) event in a gene encoding the 4-diphosphocytidyl-2-C-methyl-D-erythritol kinase (IspE). IspE participates in the methylerythritol phosphate (MEP) pathway of isoprenoid biosynthesis. The mutation in the *al4* mutant occurred at an exon-intron junction (GT-AG) of *IspE*, leading to the exon-skipping and producing a dysfunctional truncated IspE protein. The chloroplasts in the *al4* mutant are severely damaged and unable to form an intact thylakoid structure, and the mutant plants die at the three-leaf stage. This finding underscored the critical nature of splicing and alternative splicing processes, with significant repercussions on plant growth, development, and fitness.

At the biochemical level, splicing occurs *via* the formation of spliceosome complexes. The process is orchestrated by several splicing factors, auxiliary factors or co-factors, and small nuclear ribonucleoproteins (snRNPs). Golisz et al. studied the function of a core snRNP protein, *SmD3*, in plant immunity. The authors assessed the sensitivity of *A. thaliana SmD3* loss-of-function mutants (*smd3a* and *smd3b*) to *Pseudomonas syringae* pv. Tomato (Pst) DC3000 infection, as well as pathogen-associated molecular patterns such as flagellin (flg22), EF-Tu (elf18), and coronatine (COR). *smd3* mutants exhibit enhanced susceptibility to Pst accompanied by marked changes in the expression of several pathogenesis-related marker genes. Transcriptomic analysis of the s*md3-b* loss-of-function line upon pathogen infection revealed defects in splicing and altered alternative splicing patterns. Curiously, loss-of-function of *SmD3-b* also impaired stomatal development. Since stomata are critical entry points for pathogens, they propose that malfunction of the stomata could be the primary cause of the enhanced susceptibility of *smd3-b* mutant to Pst.

Splicing co-factors regulate constitutive and alternative splicing by recognizing and binding to the polypyrimidine sequence between the intron branch site and the 3′-splice sites. Lu et al. performed a comparative genomic analysis and identification of a crucial splicing co-factor, U2AF65A, among diverse plant species from algae to angiosperms. The authors identified 113 putative U2AF65A sequences from 33 plant species and performed comparisons of gene structure, protein domains, promoter motifs, and gene expression levels. Furthermore, functional studies were conducted in rice and *A. thaliana*. In rice, *U2AF65A* expression was perturbed by diverse environmental stresses, such as drought, high salinity, low temperature, and heavy metal exposure (e.g., cadmium). Loss-of-function mutant analysis in rice and *A. thaliana* revealed that U2AF65A is essential for plant tolerance to high-temperature stress and normal growth and development. Collectively, these studies presented new information on the role of alternative splicing processes and the function of snRNPs and splicing co-factors in plants in response to stress. The knowledge gained could be leveraged to improve the resilience of crops to biotic and abiotic stresses in the future.

## Author contributions

KM drafted the editorial. KM, EP, AD, and KK. provided feedback and edits to the editorial. All authors contributed to the article and approved the submitted version.

